# Use of animal models to understand titin physiology and pathology

**DOI:** 10.1111/jcmm.17533

**Published:** 2022-09-06

**Authors:** Matteo Marcello, Viviana Cetrangolo, Marco Savarese, Bjarne Udd

**Affiliations:** ^1^ PhysioLab Università di Firenze Sesto Fiorentino Italy; ^2^ Folkhälsan Research Center Helsinki Finland; ^3^ Department of Medical and Clinical Genetics Medicum, University of Helsinki Helsinki Finland; ^4^ Department of Neurology Vaasa Central Hospital Vaasa Finland

**Keywords:** animal models, congenital myopathy, dilated cardiomyopathy, mdm, medaka, mice, titin, zebrafish

## Abstract

In recent years, increasing attention has been paid to titin (*TTN*) and its mutations. Heterozygous *TTN* truncating variants (TTNtv) increase the risk of a cardiomyopathy. At the same time, TTNtv and few missense variants have been identified in patients with mainly recessive skeletal muscle diseases. The pathogenic mechanisms underlying titin‐related diseases are still partly unknown. Similarly, the titin mechanical and functional role in the muscle contraction are far from being exhaustively clarified. In the last few years, several animal models carrying variants in the titin gene have been developed and characterized to study the structural and mechanical properties of specific titin domains or to mimic patients' mutations. This review describes the main animal models so far characterized, including eight mice models and three fish models (Medaka and Zebrafish) and discusses the useful insights provided by a thorough characterization of the cell‐, tissue‐ and organism‐phenotypes in these models.

## 1 INTRODUCTION

Titin is well‐known as the largest sarcomeric protein expressed in skeletal and cardiac muscle.^1^ It plays a crucial structural and functional role in sarcomeres.^2^, ^3^, ^4^

In the last few years, an increasing number of inherited myopathies and cardiac disorders associated with titin (*TTN*) variants have been identified.^5^ These disorders differ with a very large spectrum regarding inheritance (dominant or recessive), age at onset, progression and pattern of affected muscles.^5^

Heterozygous carriers of titin‐truncating variants (TTNtv) affecting exons constitutively expressed in heart have an increased risk of adult‐onset dilated cardiomyopathy (DCM).^6^, ^7^, ^8^ A secondary genetic or non‐genetic hit is, however, usually required to develop the cardiac disease.^9^ The pathomechanism underlying titin‐related cardiomyopathies is still unclear: a reduced expression of wild‐type protein and the stable expression of truncated titin protein have been recently reported analysing myocardial tissue samples from patients with titin‐related DCM.^10^, ^11^

Biallelic mutations, mainly TTNtv with few non‐truncating variants, cause skeletal muscle diseases with or without a cardiac involvement.^5^, ^12^, ^13^, ^14^, ^15^, ^16^, ^17^, ^18^, ^19^ The increasing number of patients diagnosed with a recessive titinopathies is facilitating the delineation of genotype–phenotype correlations.^20^, ^21^ Finally, non‐truncating variants in two specific exons (344 and 364) cause distinct, well‐characterized, phenotypes (HMERF and TMD/LGMD2, respectively).^22^, ^23^, ^24^

At the same time, spontaneous and genetically engineered animal models bearing variants in the titin gene have been characterized. This review aims to describe the main animal models so far studied, including eight mice models and three fish models (Medaka and Zebrafish) (Table 1).

**TABLE 1:** Titin models described in this review

Name	Animals	Mutation	Phenotype	References
*mdm*	Mice (spontaneus)	Deletion and a LINE‐1 element integration within I‐band region (between the N2A and the proximal PEVK regions)	Muscle dystrophy	Garvey et al.^42^; Powers et al.^47^; Tahir et al.^48^; Hessel et al.^49^
*Ttn* ^*Δ30‐38*^ *(IG‐KO)*	Mice (induced)	Deletion of 9 Ig‐like domains in the proximal I‐band segment	Cardiac hypertrophy; kyphosis and smaller soleus and diaphragm muscles.	Chung et al.^50^; Buck et al.^52^
Ttn^Δ112‐158^	Mice (induced)	Deletion of 47 PEVK exons (corresponding to the human exons 112–158)	Longitudinal hypertrophy	Brynnel et al.^55^
*Ttn* ^*Δ49*^ *(N2B KO)*	Mice (induced)	Deletion of the N2B specific region	Impaired diastolic function.	Radke et al.^56^; Radke et al.^57^
*Ttn* ^*Δ219‐225*^ *(PEVK‐KO)*	Mice (induced)	Deletion of 7 PEVK exons (corresponding to human exons 219–225 or 220–226 using the current numbering)	Impaired diastolic function. Changes in muscle contractility.	Van der Pijl et al.^60^
*Ttn* ^*ΔIAjxn*^	Mice (induced)	Deletion of 14 Ig‐like and Fn3 domains (corresponding to human exons 252–270 in the current numbering) in the I‐band/A‐band (IA) junction	Exercise intolerance and a left ventricle hypertrophy.	Granzier et al.^58^
*FINmaj*	Mice (induced)	11‐bp deletion/insertion mutation in the last exon	Mild to severe muscle dystrophy. Dilated cardiomyopathy in homozygous mice.	Charton et al.^64^
ΔMex5	Mice (induced)	Deletion of second last exon (human exon 363 using the current numbering)	Muscle dystrophy and dilated cardiomyopathy.	Charton et al.^65^
*Pickwick* ^*m171*^	Zebrafish (induced)	Nonsense variant in the N2B region	Dilated cardiomyopathy	Xu et al.^71^
*runzel*	Zebrafish (induced)	Point mutation in the N2A region	Muscle dystrophy	Steffen et al.^72^
*nsh*	Medaka (spontaneus)	Point mutation in the Medaka exon 204 (ENSORLT00000022736), corresponding to human exon 349 (using the current numbering)	Hypertrophic cardiomyopathy	Higashikuse et al.^69^

## 2 TITIN: ROLE AND IMPORTANCE

Titin is giant protein that resides within the sarcomere in the striated muscle and heart.^1^ It spans the entire I‐and A‐bands of the sarcomere, connecting the Z‐disc end of the actin filament with the tip of the myosin filament, and running bound to the surface of the myosin thick filament, through the A‐band to the M‐line at the centre of the sarcomere.^2^

**FIGURE 1:**
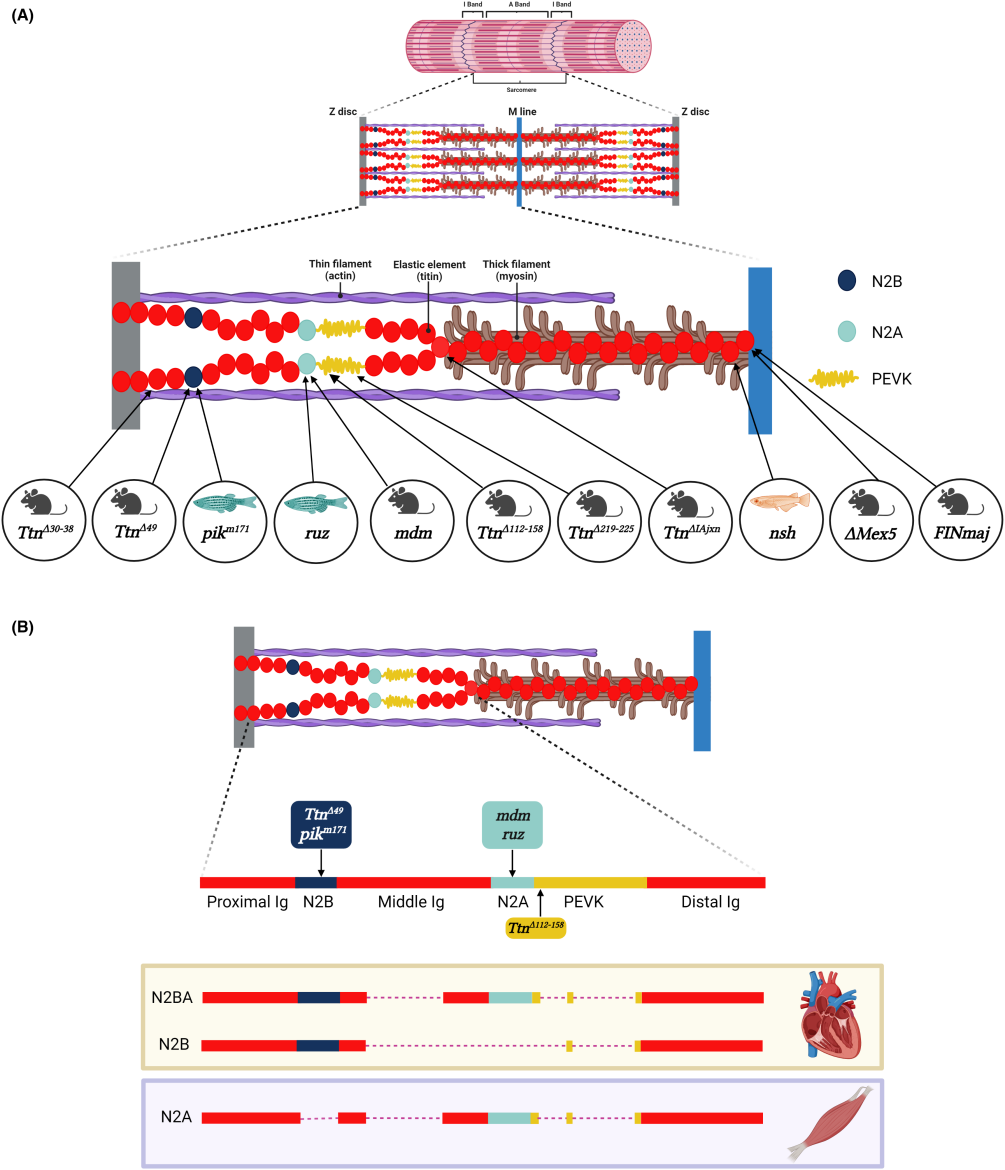
Schematic representation of titin localization into sarcomere and of titin cardiac and skeletal muscle main isoforms. (A) Two titin protein (red) spans each half‐sarcomeres, connecting the Z‐disc (grey) end of the actin thin filament (purple) with the tip of the myosin thick filament (brown), and running bound to the surface of this, up to the M‐line (blue) at the centre of the sarcomere. The Titin represented here is a theoretical isoform, in which all different domains fully expressed, to permit a correct localization in a unique figure of all the animal model mutation described in this review. (B) The main skeletal muscle isoform contains the N2A element (mutated in the mdm mice and in the ruz zebrafish model). The Titin cardiac isoforms include either the sole N2B element (mutated in the mice *Ttn* ^*Δ49*^and in the medaka *pik* ^*m171*^), or both the N2A and N2B elements. The N2B isoforms also lack part of the Middle Ig domains and include a shorter PEVK (lacking those exons deleted in the *Ttn* ^*Δ112–158*^ mice). The image is created with Biorender.

The structural role of titin in the muscle fibres is well characterized (Figure 1). Given its high number of interactions with other sarcomeric proteins, titin is a central hub that controls many of the mechanical properties of the sarcomere at rest and during contraction.^2^, ^25^ In addition, Titin is also important for sarcomere formation, mechanosensing and signal transduction.^25^, ^26^, ^27^, ^28^

The N‐terminal portion of titin interacts with several structural and signalling proteins like nebulin, α‐actinin, telethonin, actin binding proteins and filamin C.^29^, ^30^, ^31^ I‐band titin includes mainly repetitive immunoglobulin (Ig) domains and the so‐called PEVK region, which is named after the proline (P), glutamate (E), valine (V) and lysine (K) rich content. This region can extend when mechanical force is applied, providing the extensible or “spring‐like” function of titin.^1^, ^4^, ^32^ A‐band titin region is a non‐extensible region that interacts with myosins. It is composed of repetitive Ig domains and fibronectin domains. The M‐band contain the serine/threonine kinase domain, a central hub for many signalling ligands, and interacts with myomesin and obscurin originating a scaffold that link thick filaments at the M‐line of the sarcomere.^33^, ^34^, ^35^, ^36^

## 3 *TTN* GENE AND TRANSCRIPTS

Human *TTN* gene includes 364 exons (363 coding and one additional non‐coding exon).^1^, ^37^ Titin transcripts undergo alternative splicing that can theoretically produce more than 1 million isoforms.^1^, ^15^, ^38^ In particular, the I‐band region of Titin is prone to alternative splicing events, which regulate the inclusion or removal of Ig‐domains, the length of the elastic PEVK element^32^ and also the expression of two important signalling hubs, the N2A and N2B elements.^1^, ^2^, ^4^

The presence of these two elements (N2A and N2B) is likewise used to classify titin isoforms in three main categories. In particular, the N2A isoforms (containing only the N2A element) are expressed in the skeletal muscles. The two mainly cardiac isoforms are classified by the presence of only N2B element (N2B isoforms), or both the elements (N2BA isoforms).^3^, ^39^ The N2A and N2BA isoforms typically include a higher number of Ig‐domains and a longer PEVK than the N2B isoforms.

Moreover, three further isoforms (Novex‐1, Novex‐2, Novex‐3) have been reported. Novex‐1 and Novex‐2 are similar to the N2B isoforms, except for the inclusion of an isoform‐specific exon (exon 45 in Novex‐1 and exon 46 in Novex‐2). The Novex‐3 isoform exclusively contains the N‐terminal region of titin, because of an alternative stop codon in the exon 48. A recently discovered isoform, Cronos, lacks the N‐terminal and it is expressed in foetal and adult cardiac tissue.^40^, ^41^

An inferred transcript (metatranscript) including all the coding exons (except the exon 48) is also commonly used (NM_001267550.1). Several *TTN* exons from this metatranscript are not included in the aforementioned canonical post‐natal isoforms. These exons are referred to as metatranscript only exons and they are thought to be expressed during embryonic development, although some of them still have a low but detectable post‐natal expression.^14^, ^38^ Alternative splicing events (ASE) occurring in different developmental and physiological states or in anatomically different muscles result in longer or shorter titin isoforms with a variable expression.^42^, ^43^, ^44^

## 4 MUSCULAR DYSTROPHY WITH MYOSITIS (MDM)

The muscular dystrophy with myositis mice (mdm) is by far the most studied titin animal model, widely used to characterize titin physiology in skeletal muscle. This spontaneous model contains a complex rearrangement (a deletion and a LINE‐1 element integration within titin I‐band region between the N2A and the proximal PEVK regions). The rearrangement leads to an aberrantly spliced transcript and, thereby, to a protein lacking 83 amino acids including the deletion of the N2A calpain‐3 binding region.^45^ Homozygous mice (mdm/mdm) are smaller compared with their wild‐type siblings and show kyphosis, rigid and limited gait, associated with a severe muscle degeneration and necrotic fibres with phagocytosis, that had been initially mis‐interpreted as inflammation hallmarks (thereby, the name myositis). They develop an early onset progressive muscular dystrophy. Mechanical experiments have showed a significantly impaired muscle contractile function already at 6 weeks of age.^46^ The mice die at 2 months of age,^45^ due to the loss of diaphragmatic contractile function leading to respiratory failure.

The pathomechanism underlying the observed muscle phenotype has been delineated by several more recent studies. In 2016, Powers and colleagues suggested that, in psoas muscle, titin stiffness enhances the force by stabilizing the sarcomere during force development. This mechanism is lost in mdm mice in which the skipped domains in the N2A and in the proximal PEVK regions lead to a decrease in titin stiffness in an active sarcomere.^47^ More recent studies on soleus confirmed that, during activation, titin is the only known sarcomeric non‐cross bridge viscoelastic element that interacts at high affinity with Ca2+ and actin.^48^, ^49^ This interaction is mediated by the regions deleted in mdm mice.^48^, ^49^

## 5 PROXIMAL IMMUNOGLOBULIN‐LIKE DOMAINS KNOCK‐OUT (IG‐KO OR *Ttn* ^Δ30^ ^–38^)

The immunoglobulin‐like domains knock‐out mouse model (IG‐KO) has been generated deleting nine Ig‐like domains in the proximal I‐band segment of titin. Overall, 783 amino acids encoded by exons corresponding to human exons 30–38, are deleted. This model, generated by Chung and colleagues in 2013,^50^ partly recapitulates the phenotype observed in patients with heart failure with preserved ejection fraction (HFpEF). HFpEF patients and the IG‐KO model show a cardiac hypertrophy due to an increase of titin‐related diastolic stiffness.^51^ Moreover, the IG‐KO mice also display kyphosis and smaller soleus and diaphragm muscles.^52^

In these IG‐KO mice, the observed increased stiffness in heart is only due to the deletion of the nine Ig‐like domains in the proximal I‐band of titin since no compensatory changes in *TTN* cardiac isoform expression have been detected.^50^ On the contrary, in the soleus of the IG‐KO mice, the I‐band deletion seems to alter the splicing pattern of the PEVK region resulting in smaller and much stiffer isoforms. Thereby, a significant increase of passive stress and a decrease of the developed active force is observed in the IG‐KO whole soleus muscle.^52^ The noted high activity level of RBM20, a well‐known TTN splicing factor, could explain the presence of smaller isoforms due to the modified PEVK splicing pattern.^53^, ^54^

## 6 *Ttn* ^Δ112^ ^–158^ MICE

The *Ttn* ^Δ112–158^ mouse model carries a deletion of 47 PEVK exons (corresponding to the human exons 112–158). The almost 75% reduction of the PEVK segment length observed in the soleus was expected to induce an increase of the passive stiffness at the levels of the sarcomere, single fibres and whole muscle.^55^ However, a compensatory mechanism with a longitudinal hypertrophy and a consequently increased number of sarcomeres is observed. This readjustment process shortens the in vivo sarcomere length working range and thereby decreases the passive stiffness. The shortening of the thin filaments minimizes the negative effect of this readjustment on active force. The reduced weight of the Ttn^Δ112–158^ mice is probably due to the high energetic cost of the aforementioned compensatory process that increases the number of sarcomeres by nearly 30%.^55^

## 7 N2B KNOCK OUT MICE (N2B‐KO)

The N2B knock out mouse model (N2B‐KO) carries a deletion of the N2B specific exon (corresponding to the human exon 49).^56^ This single exon deletion does not alter the reading frame and leads to normal inclusion of the mutant slightly truncated protein in the sarcomere. The N2B‐KO mice survive to adulthood and are also fertile. They show a smaller heart compared to the wild‐type controls with normal ejection volumes. Decreased levels were observed of the cardiac specific protein FHL2, a member of the LIM domain gene family. Although the mice show normal systolic function, diastolic wall stress is increased and accompanied with impaired diastolic function, due to slightly reduced sarcomere length and increased passive tension of the cardiomyocytes.^56^ The increased passive tension is probably caused by the expression of the shortened titin isoform, without additional alterations in TTN expression or splicing profile.^57^

## 8 *Ttn* ^Δ219^ ^–225^ MICE OR PEVK‐KO

Seven PEVK exons (corresponding to human exons 219–225 or 220–226 using the current numbering) are deleted in the *Ttn* ^Δ219–225^ mice (also known as PEVK‐KO). These exons are constitutively expressed in the main cardiac titin isoform N2B.^58^

The PEVK region is a major source of titin elasticity in the range of the physiological sarcomere length.^59^ As expected, excision of seven exons in this region leads to an increase of the titin‐based passive tension, causing diastolic dysfunction in the adult mice. Mutant mice show both longitudinal and cross‐sectional hypertrophy due to an increased number of sarcomeres in series and in parallel. PEVK‐KO mice have hypertrophied hearts with upregulated expression of FHL proteins, proving that the dysregulation of the FHL2 levels is associated with cardiac hypertrophy.^58^

Further studies on skeletal muscle have revealed an unexpected increase (53% in soleus and 62% in EDL) of passive tension in Ttn^Δ219–225^ mutants, in contrast to the predicted increment of 17%. Co‐expression of two titin isoforms: the Ttn^Δ219–225^ N2A titin (larger isoform) and N2A2 (smaller isoform) is the likely explanation. The N2A2 isoform seems to be a splicing adaption leading to a reduced expression of elastic elements. Changes in muscle contractility have also been observed, implicating the PEVK region in the regulation of both passive and active muscle mechanics as well as of muscle plasticity.^60^

## 9 *Ttn* ^ΔIAJXN^

The *Ttn* ^ΔIAjxn^ mouse model has been generated to remove 14 immunoglobulin‐like and fibronectin type III domains (exons 251–269 corresponding to exons 252–270 in the current human numbering) located in the I‐band/A‐band (IA) junction.^61^ This titin region is not a part of the extensible spring zone of titin and the deletion does not change thick filament length. However, it moves the attachment point of the titin Ig proximal region away from the Z disk (∼65 nm). Consequently, the mice heart shows the exercise intolerance and a left ventricle hypertrophy, mimicking the heart failure with preserved ejection fraction observed in some titin patients.^61^, ^62^

## 10 FINmaj MICE

The human FINmaj mutation is a 11‐bp deletion/insertion mutation located in the last exon of *TTN*.^23^ The variant results in a 4‐amino acid exchange in the C‐terminal Ig domain M10 of the M‐band titin. FINmaj is a founder mutation in the Finnish population with a frequency close to 1/2000 individuals and causes the late onset distal myopathy named tibial muscular dystrophy (TMD).^23^

Mice carrying the FINmaj mutation well recapitulate the phenotype observed in patients.^63^ Heterozygous mice develop a late‐onset mild progressive myopathy with dystrophic changes visible from 9 months of age in three muscles, tibialis anterior, biceps femoris and quadriceps. No significant functional impairment is, however, detected. Homozygous mice show a more severe phenotype with an earlier onset (dystrophic features are visible in soleus already after 1 month of age). A dilated cardiomyopathy with heart muscle fibrosis and left ventricular dysfunction is only observed in the homozygous mice.

In patients as well as in the model mice, the mutation causes a pathological *in cis* cleavage of the last C‐terminal domains of the mutated titin protein. A calpain‐3 binding site is located in proximity in the cleaved off region and dysregulation of the proteolytic activity seems to play a crucial role in the pathology.^64^

## 11 ΔMEX5 MICE

ΔMex5 mice carry a homozygous deletion of *Ttn* second last exon (exon 363), an alternatively splicing exon encoding the insertion sequence 7 (is7).^65^ Interestingly, the exon 363 usage is highly variable in anatomically different muscles. Skeletal muscles undergoing aerobic exercises usually show a higher expression of is7+ isoforms.^38^, ^66^ Similarly, heart main isoform expresses the 363 exon.^7^ The c‐terminal part of titin interacts with numerous partners and is7 is included in a binding site for calpain 3, an enzyme almost absent in the cardiac muscle.^67^

The total absence of is7+ isoforms in the homozygous mice results in a dystrophic phenotype in those muscles where the exon is usually expressed (e.g. the soleus muscle).^65^ The is7+ isoforms are highly expressed in heart, and the is7+ sequence, encoded by exon 363, is believed to be essential for proper cardiac function in mice, explaining the observed dilated cardiomyopathy in ΔMex5 mice.^68^

Although the absence of is7 has a direct consequence only on the interaction with calpain‐3, which is not expressed in heart, it is highly probable that M‐line titin acts as a protein scaffold and, thereby, the absence of is7 may impact other interactions, partly explaining the observed pathology.

## 12 FISH MODELS (AN INNOVATIVE TOOL FOR THE INVESTIGATION OF TITIN MUTATION)

Zebrafish (*Danio rerio)* and Medaka *(Oryzias latipes)* are two fish models in which titin has a structure similar to the human one Table 2. These animal models are, therefore, emerging as alternative cheaper models to study titin function and to characterize the effect of large‐scale mutations on the cardiac and skeletal muscle phenotype. Teleost fish underwent a whole genome duplication event which led, in zebrafish, to the formation of two copies of titin, *ttna* and *ttnb*. In contrast, medaka has only one titin gene, which is thought to be composed of 219 exons and shows evolutionary highly conserved regions.^69^ Because of the evolutionary distance between medaka and zebrafish (about 110 million years ago) and the consequent subfunctionalization of gene copies, the repertoire of the duplicated genes is different, and the two models are unlikely to show redundant phenotypes. Thus, the medaka fish model, due of its reduced genomic duplication and unique phenotypes attributable to specific mutations, is a promising model for future studies of titin mutations.

**TABLE 2:** Other zebrafish titin models (described in Santiago et al.^70^)

Name	Gene	Mutation Location
*ttna_N2A, ttna_N2B, ttnb_N2A and ttnb_N2B morphants (MO)*	*ttna, ttnb*	N2A, N2B
*Herzschlag (Hel* ^*tg287*^ *)*	*ttna*	I/A junction
*n1, n2, n3*	*ttna*	Z‐disc, proximal I‐band, mid‐I band
*c1, c2, c3*	*ttna*	Proximal, mid and distal A band
*Ttn.2* ^*xu064*^	*ttna*	Z‐disc
*Ttn.2* ^*xu065*^	*ttna*	A‐band
*Ttn.1* ^*xu066*^	*ttnb*	Z‐disc
*Ttn.1* ^*xu067*^	*ttnb*	A‐band
*ttn* ^*xu068*^	*ttna*	Z‐disc & A‐band
*ttn* ^*xu069*^	*ttna ttnb*	Z‐disc
*ttn* ^*xu070*^	*ttna ttnb*	A‐band
*ttn* ^*xu071*^	*ttna ttnb*	Z‐disc A‐band
*ttna* ^*tv*^	*ttna*	A‐band

A recent review published by Celine F. Santiago et al.^70^ has discussed in depth fifteen different zebrafish titin mutated models created to characterize the cardiac structure and function and to study genetic and molecular mechanisms in muscle disease (Table 2). This was possible thanks to new tools of imaging and “‐omics” technologies for the cardiac phenotyping in small animal models. Conversely, to date just one medaka model has been reported with a titin mutation. Herein, we report two zebrafish models, which carry titin mutation linked to a cardiac (*Pickwick* ^*m171*^ *)* and a skeletal (*runzel*) myopathy, and the medaka cardiac model *nsh*.

## 13 ZEBRAFISH TITIN MODELS

### 13.1 Pickwick^m171^

*Pickwick* ^*m171*^ *(pik* ^*m171*^) is a recessive lethal mutation discovered in zebrafish during a large‐scale genetic screen.^71^ The mutation found was a T > G transversion, located in the unique sequence of the cardiac N2B exon. The *pik* ^*m171*^ heart develops normally during the first 3 days but is poorly contractile due to abnormal sarcomeres.^71^ This mutation in the N2B exon causes the generation of a stop codon and a resulting truncated protein devoid of N2B and M‐line regions. The consequence is sarcomeric disassembly with thinner myofibrils and impaired systolic function. Using a morpholino approach to confirm the genotype–phenotype relation, Xu and colleagues disrupted the expression of the cardiac N2B exon in morphants, which led to the same phenotype as the *pik* ^*m171*^mutants.^71^ These results indicate the requirement of titin for the correct assembly of the sarcomeres and resembles the cardiac phenotype of patients with dilated cardiomyopathy (DCM).^71^

### 13.2 Runzel

*Runzel (ruz)* is a mutation in the N2A region, isolated after the n‐ethyl‐n‐nitrosourea (ENU) screening, and causing a recessive muscle dystrophy.^72^ *ruz* zebrafish mutants show decreased muscle organization already at 3 days post fertilization (dpf) and low mobility with reduced swimming ability at 5–7 dpf, with a general sarcomeric disassembly and poorly organized myofibrils.^72^ While heterozygotes did not show any abnormalities in mobility, in homozygosity this mutation appears to be lethal. Morpholino injections produced a phenocopy of the *ruz* mutant and, together with RNA and protein analyses in *ruz* mutants, confirmed that the *ruz* mutation is located in the skeletal titin locus. Also, *ruz* mutants displayed a progressive reduction of titin isoforms throughout developmental stages highlighting the lack of titin isoforms switching.^72^

## 14 TITIN *NSH* MEDAKA

A point mutation causing an abnormal heart phenotype was reported after an n‐ethyl‐n‐nitrosourea (ENU)‐induced mutation screening in medaka *Oryzyas latipes*.^69^ The mutant was named *non‐spring heart (nsh)*. Positional cloning placed the point mutation in the exon 204 (ENSORLT00000022736, corresponding to human exon 349), that encodes an Ig‐domain near the MURF (muscle‐specific ring finger protein 1)‐binding region. The *nsh‐*homozygote embryos survived up to 8 days post fertilization (dpf) but, eventually, they did not hatch.^69^ These embryos displayed hypertrophic myocardial walls, which caused slow or clogged blood flow. The cardiomyocytes showed loose arrangement of the contractile filaments, broken thick and thin filaments and non‐paralleled Z‐discs. Also, *nsh* homozygotes displayed a smaller body, in which the skeletal muscles had an abnormal sarcomeric structure with disrupted myofibrils. On the molecular level, these homozygote mutants exhibit differential expression of the cardiac stretch marker *bnp* (higher in the ventriculum and lower the atrium) compared with the wild‐type controls and an increased proportion of N2B isoforms probably causing less elasticity and increased passive stiffness of the cardiac myofibrils. Furthermore, the *nsh* adult heterozygotes showed M‐line disassembly in myofibrils linked to abnormal sarcomeric structures. Nevertheless, their size, movement, lifespan and fertility were not affected. Both these phenotypes are due to the Ig‐domain missense mutation located in the transition zone between the A‐band and the M‐line of titin, which leads to an increased binding of titin to MURF1 and an enhanced titin degradation by ubiquitination. Similar phenotypes with M‐line disassembly were found in hypertrophic cardiomyopathy (HCM) patients also associated with *TTN* mutations in the A‐band/M‐line transition zone. Thus, the impaired interaction of titin and MURF1 highlights a novel mechanism associated with the pathogenesis of HCM and suggests inhibition of MURF1 function as a possible therapy for HCM patients.^69^

## 15 CONCLUSIONS AND PERSPECTIVES

To date, most TTN animal models have been developed to characterize the structural and mechanical properties of specific titin domains that are physiologically or clinically relevant. Except the FINmaj mouse (recapitulating the human TMD/LGMD R10 phenotypes) and the *nsh* Medaka models (carrying a missense variant also found in patients),^63^, ^69^ the genetic defects in the remaining animal models do not mirror the mutations identified in titinopathy patients so far. However, the thorough characterization of the cell‐, tissue‐ and organism‐phenotypes in these models has provided very useful insights:

1. Even large heterozygous but, probably, even homozygous in‐frame deletions of titin specific regions are relatively well tolerated, although still resulting in a cardiac and/or skeletal muscle phenotype somehow resembling the observed *TTN*‐related clinical entities;
2. These large deletions often activate a compensatory mechanism resulting in the expression of shorter and stiffer isoforms;
3. The main titin splicing factor, RBM20, could be a potential therapeutic target and its downregulation could lead to the expression of longer and more compliant titin isoforms (reverting the mechanism induced by the large deletions).

New animal models carrying mutations resembling those found in patients would be useful to characterize the pathomechanisms underlying human diseases.

At the same time, skinned fibres from patients´ muscle biopsies could be used for mechanical experiments although technical aspects (e.g. sample collection, amount and quality of patients‐derived material, the need for healthy sex‐ and aged‐matched control muscles) may hamper a relevant outcome of these experiments.

Finally, 3D culture models from human cells are emerging as a potential alternative approach other than vertebrate model organisms.^73^ 3D cultures mimic the structure and the functionality of human muscle tissues. Although currently the physiological properties of the 3D cultures are not yet comparable with those found in vivo in tissues, they may soon become a very useful tool for muscle disease modelling.^74^ Since each of these approaches presents issues that can compromise its effectiveness, their synergistic use could be optimal to understand how a specific titin mutation leads to the development of a given clinical phenotype.

## AUTHOR CONTRIBUTIONS

**Matteo Marcello:** Conceptualization (equal); data curation (equal); writing – original draft (lead); writing – review and editing (equal). **Viviana Cetrangolo:** Data curation (equal); writing – original draft (equal); writing – review and editing (supporting). **Marco Savarese:** Conceptualization (equal); data curation (equal); funding acquisition (equal); writing – original draft (supporting); writing – review and editing (lead). **Bjarne Udd:** Conceptualization (equal); funding acquisition (equal); writing – review and editing (equal).

## FUNDING INFORMATION

M.S. work is supported by *Academy of Finland* (grant #339437 ´Improving the clinical interpretation of sequence variants´) and *Sydäntutkimussäätiö* (´Titin truncating variants and dilated cardiomyopathy´). B.U. work is supported by *Academy of Finland* and *European Joint Programme on Rare Diseases* (‘Improved diagnostic output in large sarcomeric genes IDOLS‐G’).

## CONFLICT OF INTEREST

The authors declare no competing interests.

## Data Availability

Data sharing is not applicable to this article as no new data were created or analyzed in this study.
